# Childhood immunization rates in rural Intibucá, Honduras: an analysis of a local database tool and community health center records for assessing and improving vaccine coverage

**DOI:** 10.1186/1471-2458-12-1056

**Published:** 2012-12-07

**Authors:** Yuan He, Alan Zarychta, Joseph B Ranz, Mary Carroll, Lori M Singleton, Paria M Wilson, Elizabeth P Schlaudecker

**Affiliations:** 1 Shoulder to Shoulder, Concepción, Intibucá, Honduras; 2 Global Health Center, Cincinnati Children’s Hospital and Medical Center, Cincinnati, OH, USA; 3 Global Health Track, Pediatric Residency Training Program, Cincinnati Children’s Hospital and Medical Center, Cincinnati, OH, USA; 4 Division of Infectious Diseases, Global Health Center, Cincinnati Children’s Hospital and Medical Center, Cincinnati, OH, USA

**Keywords:** Vaccines, Childhood immunization, Honduras, Database, Community health workers, Public health

## Abstract

**Background:**

Vaccines are highly effective at preventing infectious diseases in children, and prevention is especially important in resource-limited countries where treatment is difficult to access. In Honduras, the World Health Organization (WHO) reports very high immunization rates in children. To determine whether or not these estimates accurately depict the immunization coverage in non-urban regions of the country, we compared the WHO data to immunization rates obtained from a local database tool and community health center records in rural Intibucá, Honduras.

**Methods:**

We used data from two sources to comprehensively evaluate immunization rates in the area: 1) census data from a local database and 2) immunization data collected at health centers. We compared these rates using logistic regression, and we compared them to publicly available WHO-reported estimates using confidence interval inclusion.

**Results:**

We found that mean immunization rates for each vaccine were high (range 84.4 to 98.8 percent), but rates recorded at the health centers were significantly higher than those reported from the census data (*p*≤0.001). Combining the results from both databases, the mean rates of four out of five vaccines were less than WHO-reported rates (*p* <0.05). Overall immunization rates were significantly different between townships (*p*=0.03). The rates by individual vaccine were similar across townships (*p* >0.05), except for diphtheria/tetanus/pertussis vaccine (*p*=0.02) and oral polio vaccine (*p* <0.01).

**Conclusions:**

Immunization rates in Honduras were high across data sources, though most of the rates recorded in rural Honduras were less than WHO-reported rates. Despite geographical difficulties and barriers to access, the local database and Honduran community health workers have developed a thorough system for ensuring that children receive their immunizations on time. The successful integration of community health workers and a database within the Honduran decentralized health system may serve as a model for other immunization programs in resource-limited countries where health care is less accessible.

## Background

Vaccines play an important role in preventative medicine, as they are a cost-effective way to reduce the burden of infectious diseases worldwide. Vaccines have had a greater impact on childhood mortality than any other intervention, particularly in remote regions of resource-limited countries with minimal access to health care [1]. However, previous studies in rural settings have demonstrated lower immunization coverage in areas with difficult terrain and long distances between places of residence and immunization services [2].

According to data collected by the World Health Organization (WHO) [3], the Republic of Honduras has high childhood immunization rates. Official country reports estimate that over 97 percent of one year old children are immunized with the measles, diphtheria/pertussis/tetanus (DPT), hepatitis B, *Haemophilus influenzae* type b (Hib), Bacille Calmette Guérin (BCG), and polio vaccines [3]. These six immunizations, when administered in timely and appropriate dosages, help prevent these serious illnesses. Unfortunately, the estimates provided by the WHO do not always capture or take into consideration the more remote regions of the country, as it is difficult to obtain childhood vaccine and death records in rural areas without adequate vital registration systems [2,4]. Given the limited access to health care and difficulties of data collection, immunization records and rates may be less comprehensive in these areas.

Hombro a Hombro/Shoulder to Shoulder is a non-governmental organization that has served rural Honduras for over 20 years. The organization has recently collected census data for the municipality of Concepción, which includes immunization information for children less than 5 years of age. This study aims to assess the effectiveness of using Shoulder to Shoulder’s local database and community health workers to analyze current immunization coverage within the region by comparing them to WHO-reported estimates [3]. The results of this analysis may guide future interventions to improve rates and coverage, thereby decreasing the number of children at risk for preventable diseases.

## Methods

### Census area

Intibucá is one of the 18 states (“departments”) into which the nation of Honduras is divided. Concepción is one of 16 municipalities in the department, with a population of approximately 10,458 people [5]. The nearest hospital is located in La Esperanza, the capital of Intibucá, approximately 35 kilometers away or three hours by bus. The mountainous terrain and limited transportation hinder access to care for Hondurans in this region.

Community health workers and data analysis staff hired by Shoulder to Shoulder conducted the census from November of 2009 to April of 2010 using a standardized form. Shoulder to Shoulder staff traveled from home to home collecting vital registration information such as births, deaths, and geographic location, as well as supplemental health information, including immunization information for each child in the home recorded on vaccination cards. These vaccination cards are assigned to individual children, and the parent is responsible for bringing the card to the health center at the time of immunization. Township divisions are slightly different in our two data sources; certain communities are included in one township in the Shoulder to Shoulder database, but another township according to the Honduran government. For the purposes of this analysis, we used Shoulder to Shoulder township boundaries.

### Shoulder to Shoulder database

The data used for this study are not openly available; we received permission to use vaccine data for individuals in rural western Honduras from Shoulder to Shoulder, the custodian of the relevant health records. Data analysis staff systematically entered census information into Shoulder to Shoulder’s database, which organizes health and demographic information for quick access and updates. The Shoulder to Shoulder database system is written in Microsoft Access 2007. The system interface is via 30 forms that are supported by approximately 150,000 lines of Visual Basic Code, which provide navigation, error checking and selected context sensitive help. The database is structured around eight main tables and a series of support tables. Shoulder to Shoulder employs six to eight full-time Honduran staff members dedicated to the database. They update the database daily with new patient information, and Shoulder to Shoulder uses these data to perform analyses that direct community interventions.

### Immunization data from the Shoulder to Shoulder database

Immunization data were obtained from immunization cards during the census period and extracted from the Shoulder to Shoulder database. A database query was performed to produce an immunization table restricted to children less than 5 years of age (as of May 1, 2010) who live in the nine townships of Concepción: Calucica, Colomarigua, Concepción, El Guachipilincito, El Guajiniquil, El Rodeo, Jiquinlaca, San Nicolás, and Santiago. From this table, 14 children were found to be missing immunization records. These children were noted for subsequent data collection endeavors, but excluded from this analysis.

In order to assess the number of children who had received the accurate doses of each vaccine, a reference table was constructed to specify the appropriate dosage at each age. Because all data pertained to children less than 5 years of age, the analysis was restricted to the five vaccines administered to this age group: BCG, pentavalent (diphtheria, tetanus, whooping cough, hepatitis B, and Hib), OPV (oral polio vaccine), MMR (measles, mumps, and rubella), and DPT (diphtheria, pertussis, and tetanus booster). A second reference table was created to organize the immunization data for each child by location (department, township, and village), carnet number (a number assigned by Shoulder to Shoulder to each person registered in the database), gender, name, date of birth, age in years, and received doses of BCG, pentavalent, OPV, MMR, and DPT.

A third table cross referenced data from the reference tables to produce raw results. By creating a formula that pinpointed the number of doses the child received, and then subtracting the number of doses he or she should have received according to the first reference table, a fourth table of missing doses was constructed, organized, and sorted by township.

A final table used township population size and dosage information from the second table to calculate immunization rates and coverage. We did this by restricting the count to a single township and excluding children for whom we had no immunization data. For each percentage, the denominator was the total number of children per township.

### Immunization data from health center records

Through interviews with Shoulder to Shoulder community health workers, data analysis staff, and nurses, we discovered that the immunization cards were not always accurate or up-to-date, as they were contingent upon mothers bringing the cards in to be updated with every vaccine dose. We therefore utilized a second source of data: immunization records kept by the health centers in their “*Listados de Niños para Vigilancia Integral*” (LINVIs), or “Lists of Children for Integrated Monitoring.” We again received permission to use these vaccine data for individuals in rural western Honduras from Shoulder to Shoulder, the administrator of these health records. LINVIs are handwritten tables of immunization information kept within the three local health centers servicing the townships. The townships are divided into villages. Each LINVI contains all children less than 5 years of age who live within a specific village, organized by mother’s name and birth date. A nurse manages and updates a separate LINVI for each village serviced by the health center by recording vaccine dates. By counting the number of times a child received a vaccine by a certain date, we could estimate the received and missing doses for each child.

We collected data in November 2010 by copying all LINVI entries dating back to 2005 into a spreadsheet containing columns for: name of the village and township, name of child, name of mother, date of birth, and number of doses received of BCG, pentavalent, OPV, MMR, and DPT vaccines. Since LINVI entries did not include the carnet numbers of each child, names or dates of birth were used to identify and match children with those recorded in the database. We then applied the same calculations to the raw data collected at the three health centers. Our data entry staff used matched records to add additional and missing information to our database, including names of newborns, names of mothers, changes in location, and deaths. Children who were not found in the database were noted and copied into a new spreadsheet to be assigned a carnet number and entered into the database.

### Immunization data from Shoulder to Shoulder database and health center records

In a final assessment, we combined data from the database with data from the LINVIs, in order to create a more accurate and comprehensive table of immunization data. For children who overlapped and were present in both data sets, the most updated information was used. If a child received one oral polio vaccine according to a LINVI but three according to the database, we recorded three doses. We removed duplicate records in the cases of children who were present in two or more LINVIs, since this typically indicated children had moved to a different community.

### Statistical analyses

The joint effects of database, township, and vaccine on percent immunization were evaluated by logistic regression. The main effects of each factor and all two-way interactions were modeled. F-statistics were generated for the following effects: (1) database differences overall, in each township, and for each vaccine; (2) township differences overall, from each database, and for each vaccine; and (3) vaccine differences overall, in each township, and between databases. We obtained publicly available immunization percentages from the WHO [3] and compared these to mean immunization percentages across databases and townships for each vaccine using confidence interval inclusion. The analyses were implemented using SAS PROC GLIMMIX. An alpha level of 0.05 denoted statistical significance.

## Results

Including data from nine townships and averaging rates for each vaccine by data source, immunization coverage rates ranged from 84.4 to 98.8 percent. The rates obtained from the LINVIs were significantly higher than the rates obtained from the Shoulder to Shoulder database (*p*≤0.001), with the exception of El Rodeo (*p*=0.87). The WHO reports national averages of 97 percent or higher for all vaccines [3]. Combining the results from LINVI and Shoulder to Shoulder databases, the mean rates of four out of five vaccines were less than WHO-reported rates (*p* <0.05). Overall immunization rates were significantly different between townships (*p*=0.03). The rates by vaccine were similar across townships (*p* >0.05), except for diphtheria/tetanus/pertussis vaccine (*p*=0.02) and oral polio vaccine (*p* <0.01).

### Immunization data from the Shoulder to Shoulder database

According to the first analysis (see Table 1), the childhood immunizations with the lowest rates were the pentavalent, OPV, and DPT vaccines. The pentavalent and OPV vaccines had the highest number of outstanding doses; almost fifteen percent of children were missing one or more doses in the municipality of Concepción. Certain townships had higher immunization rates than others; Colomarigua, El Rodeo, and San Nicolás had about 90 percent coverage of all vaccines for children less than 5 years old, whereas Calucica and El Guachipilincito only covered around 70 percent of children with the OPV vaccine. Despite these differences, the overall immunization rates obtained from the Shoulder to Shoulder database were statistically similar between townships (*p*=0.11).

**Table 1:** Percent immunization coverage according to Shoulder to Shoulder database – Concepción by township

**Township**	**Population ( <5 yo)**	**BCG**	**Pentavalent**	**OPV**	**MMR**	**DPT**
Calucica	47	97.87%	87.23%	63.83%	95.74%	91.49%
Colomarigua o El Tablón	65	98.46%	90.77%	93.85%	98.46%	89.23%
Concepción	116	97.41%	84.48%	91.38%	92.24%	86.21%
El Guachipilincito	52	96.15%	82.69%	73.08%	100.00%	92.31%
El Guajiniquil	95	98.95%	88.42%	85.26%	96.84%	83.16%
El Rodeo	84	98.81%	85.71%	92.86%	95.24%	96.43%
Jiquinlaca	201	99.50%	84.58%	82.09%	92.54%	85.07%
San Nicolás	89	100.00%	91.01%	92.13%	97.75%	91.01%
Santiago	112	99.11%	87.50%	84.82%	93.75%	92.86%
**Total**	861	98.72%	86.64%	85.48%	95.01%	88.85%

Percent immunization coverage of BCG, pentavalent, OPV, MMR, and DPT vaccines by township recorded in the Shoulder to Shoulder database – according to vaccination cards carried by mothers at the time of the census.

### Immunization data from health center records

From the first analysis of census data and general observations in the health centers, it was observed that the immunization cards presented by mothers during the May 2010 census were not the most updated or accurate source of vaccination records. Compared to WHO data reporting immunization rates [3], the rates recorded in the Shoulder to Shoulder database were lower for all vaccines.

In comparison, we found that LINVI vaccine data in Concepción (see Table 2) captured more children and documented more vaccine doses than the Shoulder to Shoulder database. The rates obtained from the LINVIs were significantly higher than the rates obtained from the Shoulder to Shoulder database (*p*≤0.001), with the exception of El Rodeo (*p*=0.87). Compared to the analysis using census information in the database, there were many more children captured, and fewer of them were missing vaccine doses.

**Table 2:** Percent immunization coverage according to health center LINVIs - Concepción by Township

**Township**	**Population ( <5 yo)**	**BCG**	**Pentavalent**	**OPV**	**MMR**	**DPT**
Calucica	82	100.00%	98.78%	98.78%	96.34%	71.95%
Colomarigua o El Tablón	55	100.00%	96.36%	96.36%	98.18%	100.00%
Concepción	154	100.00%	100.00%	100.00%	100.00%	83.12%
El Guachipilincito	83	100.00%	97.59%	97.59%	97.59%	81.93%
El Guajiniquil	136	99.26%	98.53%	98.53%	99.26%	93.38%
El Rodeo	18	100.00%	100.00%	100.00%	100.00%	83.33%
Jiquinlaca	262	97.71%	97.71%	97.33%	100.00%	84.73%
San Nicolás	110	98.18%	98.18%	98.18%	100.00%	95.45%
Santiago	139	100.00%	97.84%	97.84%	99.28%	83.45%
**Total**	1039	99.13%	98.27%	98.17%	99.23%	86.14%

Percent immunization coverage of BCG, pentavalent, OPV, MMR, or DPT vaccines by township – data collected from LINVIs from the three health centers in the municipality.

### Immunization data from Shoulder to Shoulder database and health center records

Compared to the previous two analyses, the combination of vaccine cards and local health center data resulted in a much higher rate of coverage within the municipality of Concepción (see Table 3). With the exception of the third dose of OPV in one township, over 90 percent of all “captured” children less than 5 years of age received the required immunizations. Despite this improvement, the mean rates of four out of five vaccines were still significantly less than WHO-reported rates (*p* <0.05).

**Table 3:** L**ocal database coverage combined with government LINVI coverage – Concepción by township**

**Township**	**Population ( <5 yo)**	**BCG**	**Pentavalent**	**OPV**	**MMR**	**DPT**
Calucica	77	100.00%	98.70%	97.40%	96.10%	90.91%
Colomarigua o El Tablón	74	100.00%	94.59%	97.30%	98.65%	97.30%
Concepción	159	99.37%	99.37%	100.00%	99.37%	94.97%
El Guachipilincito	81	98.77%	96.30%	96.30%	97.53%	95.06%
El Guajiniquil	133	99.25%	98.50%	97.74%	99.25%	96.99%
El Rodeo	87	98.85%	86.21%	93.10%	97.70%	96.55%
Jiquinlaca	276	97.46%	96.38%	95.65%	98.91%	93.48%
San Nicolás	111	98.20%	98.20%	98.20%	100.00%	97.30%
Santiago	146	100.00%	96.58%	97.26%	98.63%	93.15%
**Total**	1144	98.86%	96.50%	97.03%	98.69%	94.84%

Percent immunization coverage of BCG, pentavalent, OPV, MMR, and DPT vaccines by township – numbers calculated from the Shoulder to Shoulder database and LINVI data.

Across all townships studied, overall immunization rates remained greater than 85 percent. Immunization rates were statistically similar across townships, except for diphtheria/tetanus/pertussis vaccine (*p*=0.02) and oral polio vaccine (*p* <0.01).

These vaccines require multiple doses at several ages and tended to have lower rates than those of BCG and MMR. BCG and MMR are both single-dose immunizations and are administered at birth and at one year, respectively.

## Discussion

These results demonstrated high immunization coverage in this remote Honduran region, though the rates are significantly lower than WHO-reported estimates. These data suggest that the broadest and most comprehensive assessment of immunization coverage in rural Intibucá, Honduras, is obtained from combining records from the Shoulder to Shoulder database with vaccination records from the local health centers. In similar rural settings, previous studies have demonstrated lower immunization coverage in areas with difficult terrain and long distances between place of residence and immunization services [2,4]. Based on our analyses, the current system appears to have had a positive impact on the frequently-cited barriers to immunization in rural settings, including parent forgetfulness and convenience of time/place for vaccinations [4]. Community health workers and other staff in Intibucá have noted these barriers and included programs such as education on vaccine importance. Vaccine delivery rates are also included in the monthly evaluation of health centers and staff by the health department. The current system of community health workers, local health centers, and home visits for promoting, delivering, and monitoring immunizations in this area has been successful, even in one of the most remote regions of Intibucá.

The comparison between immunization data from the Shoulder to Shoulder database and health center LINVIs allowed a deeper analysis of their respective advantages and disadvantages. LINVI records captured more children and tended to reflect higher vaccine dosages, but may have been more prone to error, as delivered vaccine doses were counted manually for each child. In addition, children who move among various townships may not have updated records in any LINVI, thus accounting for outstanding or extraneous doses applied. The Shoulder to Shoulder database provides an easy and effective tool for mining information, making data extraction and compilation easier and less prone to human error. However, immunization data were less up-to-date in the Shoulder to Shoulder database, since they were only as current as the date of the census. Certain cases demonstrated that the database had more immunization information than the respective LINVI, indicating that vaccine cards kept by mothers could be a more complete source of information than data assembled from various LINVIs. The immunization cards may capture more vaccine doses than the LINVI, because families often move between different communities.

Using the combined rates from both databases, there were significant differences in immunization rates between townships (*p*=0.03). El Rodeo and El Guajiniquil had lower immunization coverage for certain vaccines, which could potentially be attributed to poor recordkeeping by the nurse in the health center or poor communication between nurses and community health workers. Comprehensive LINVI records were not available for all villages of El Rodeo. Better follow-up and data tracking will help to address lower immunization rates in these areas.

There were several limitations to this study. Even though Shoulder to Shoulder serves a large catchment area, there are record-keeping differences among health centers. We were unable to determine whether lower immunization rates resulted from poor vaccine delivery or failed record-keeping. Specifically, lower OPV and DPT vaccination rates may reflect the fact that boosters of these vaccines are recorded differently in the different health centers, resulting in inaccurate counting and subsequent discrepancies in data. In addition, immunization data from the database was restricted to the one-time census, preventing real-time comparison with LINVI records. In combining the two data sets, we were able to capture a larger population, but we cannot be sure that this accurately represents the current population. There may be outdated records of children who are no longer in the municipality. This study was also limited by the lack of a comparison group. Further studies are needed to assess immunization coverage in rural areas not covered by the Shoulder to Shoulder health care system.

As a direct result of this analysis, a system was implemented to expand coverage and surveillance of immunization records in all municipalities of Shoulder to Shoulder’s catchment area. For each of the nine government health centers, data entry staff will copy LINVI records every three months to update immunization data in the database. With the LINVIs, we will be able to keep track of births, deaths, immunizations, and movement among communities. We hope that we will be able to obtain a more accurate idea of gaps in immunization coverage and their causes to improve immunization rates and record-keeping.

## Conclusions

These data suggest that the most comprehensive assessment of immunization coverage in rural Intibucá, Honduras, is obtained from combining records from the Shoulder to Shoulder database with immunization records from the local health centers. These results demonstrate very good immunization coverage in this region, though rates are still less than WHO-reported estimates. The successful use of community health workers and a local database within the Honduran decentralized health system may serve as a model for other immunization programs in countries where health care resources are limited.

## Abbreviations

BCG: Bacille Calmette-Guérin vaccine; DPT: Diphtheria-Tetanus-Pertussis vaccine; Hib: Haemophilius influenza type B vaccine; LINVI: *Listado de Niños para Vigilancia Integral* or List of Children for Integrated Monitoring; MMR: Measles Mumps, and Rubella vaccine; OPV: Sabin Oral Polio Vaccine; WHO: World Health Organization.

## Competing interests

The authors report no conflicts of interest related to this manuscript.

## Authors’ contributions

YH collected data and assisted in data analysis and manuscript writing. AZ developed and oversaw the data analysis as well as data quality management. JR contributed to data analysis and manuscript writing. MC, LMS and PMW assisted in data collection and manuscript writing. EPS oversaw the study design and contributed to data analysis and manuscript writing. This manuscript was drafted by YH and all the authors contributed to its revision and approved the final version.

## Authors’ information

YH, AZ, and JR are research assistants at the Shoulder to Shoulder Maternal and Child Health Care Center, Concepción, Intibucá, Honduras. MC, LMS and PMW are pediatric residents at Cincinnati Children’s Hospital Medical Center in the Global Health Track of the Pediatric Residency Training Program. EPS is an Assistant Professor of Pediatrics at Cincinnati Children’s Hospital Medical Center in the Global Health Center and the Division of Infectious Diseases.

## Pre-publication history

The pre-publication history for this paper can be accessed here:

http://www.biomedcentral.com/1471-2458/12/1056/prepub
